# Comparison of clinicopathological characteristics and long-term survival between patients with gallbladder adenosquamous carcinoma and pure gallbladder adenocarcinoma after curative-intent surgery: a single-center experience in China and a meta-analysis

**DOI:** 10.3389/fonc.2023.1116275

**Published:** 2023-05-02

**Authors:** Tian-Run Lv, Fei Liu, Zuo-Yu Liang, Rui-Qi Zou, Wen-Jie Ma, Hai-Jie Hu, Fu-Yu Li

**Affiliations:** ^1^Division of Biliary Tract Surgery, Department of General Surgery, West China Hospital, Sichuan University, Chengdu, Sichuan, China; ^2^Department of Pathology, West China Hospital, Sichuan University, Chengdu, Sichuan, China

**Keywords:** gallbladder, adeno-squamous, squamous, carcinoma, cancer

## Abstract

**Objective:**

The aim of the study was to evaluate the similarities and differences between gallbladder adenosquamous carcinoma (GBASC) and pure gallbladder adenocarcinoma (GBAC).

**Methods:**

Patients with GBASC and GBAC from 2010 to 2020 were analyzed in terms of clinicopathological features and long-term survival. Moreover, a meta-analysis was also performed for further validation.

**Results:**

*Our experience:* A total of 304 patients with resected GBC were identified, including 34 patients with GBASC and 270 patients with GBAC. Patients with GBASC had a significantly higher preoperative CA199 level (P <0.0001), a significantly higher incidence of liver invasion (P <0.0001), a relatively larger tumor size (P = 0.060), and a significantly higher proportion of patients with T3–4 (P <0.0001) or III–IV disease (P = 0.003). A comparable R0 rate was obtained between two groups (P = 0.328). A significantly worse overall survival (OS) (P = 0.0002) or disease-free survival (DFS) (P = 0.0002) was observed in the GBASC. After propensity score matching, comparable OS (P = 0.9093) and DFS (P = 0.1494) were obtained. Clear margin (P = 0.001), node metastasis (P <0.0001), T stage (P <0.0001), and postoperative adjuvant chemoradiotherapy (P <0.0001) were independent prognostic factors for OS for the entire cohort. Adjuvant chemoradiotherapy had a survival benefit for patients with GBAC, while the survival benefit was still being validated in patients with GBASC. *Meta-analysis:* With our cohort incorporated, a total of seven studies involving 1,434 patients with GBASC/squamous carcinoma (SC) were identified. GBASC/SC shared a worse prognosis (P <0.00001) and more aggressive tumor biological features than GBAC.

**Conclusion:**

GBASC/SC shared more aggressive tumor biological features and a much worse prognosis than those with pure GBAC.

## Introduction

Gallbladder carcinoma (GBC) is a rare and deadly biliary epithelial-derived malignancy that is the most common biliary tract cancer and the fifth most common disease of the digestive system (1, 2). Curative surgery provides the only chance of curing the disease, and a significantly improved prognosis was often observed in those who received radical resection (3). Apart from the impact of curative surgery on the overall prognosis, various factors, especially tumor histological subtypes, may also affect the prognosis to some extent.

According to the World Health Organization (WHO) classification criteria for digestive cancers (2010 edition), adenocarcinoma (AC) and squamous carcinoma (SC) account for most cases and are regarded as the major pathological subtypes. Adenosquamous carcinoma (ASC) was another rare pathology that shared extremely different tumor biological features and long-term survival with AC and SC (4). Currently, according to the WHO diagnostic criteria for ASC, except for pancreatic ASC, where the proportion of squamous components is required for more than 30% to reach a diagnosis, there is no consensus on the percentage of squamous components in various solid cancers, especially in GBC (5). Compared with the high frequency of gallbladder adenocarcinoma (GABC) (90%), gallbladder adenosquamous carcinoma (GBASC), or gallbladder squamous carcinoma (SC) can only be detected with a significantly lower incidence ranging from 2% to 10% (6). Various studies have explored the tumor’s biological features and the long-term survival of patients with GBASC/SC. However, owing to its rarity, most of them (7–10) only included a small sample size, and the relevant date regarding the Asian population is quite rare. Only two studies with a relatively small cohort reported GBASC in the Asian population (9, 10).

Therefore, the aim of our study was to evaluate the consistencies and inconsistencies between GBASC and conventionally pure GBAC with our own single-center experience in China. Moreover, with our own results incorporated, a meta-analysis was also performed to provide a more comprehensive validation.

## Patients and methods

### Patient identification

**Figure 1:**
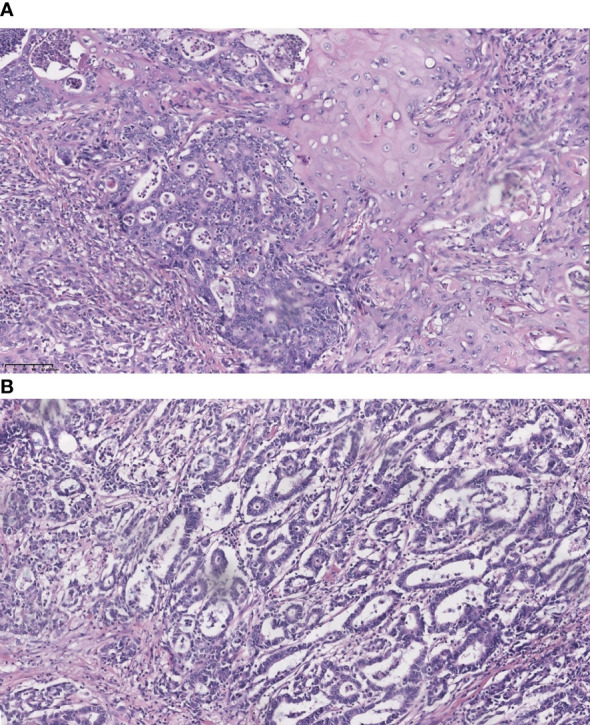
Representative section of adenosquamous carcinoma and pure adenocarcinoma of the gallbladder (hematoxylin and eosin stain, 200). **(A)** co-existence of squamous cell carcinoma and adenocarcinoma; and **(B)** pure adenocarcinoma.

We retrospectively analyzed patients who received curative-intent radical cholecystectomy at West China Hospital, Sichuan University, between September 2010 and September 2020 from a retrospective electronic database. Having ruled out patients who had lost follow-up as well as those without adequate clinical data, only patients with pathologically confirmed GBASC (**Figure 1A**) and pure GBAC (**Figure 1B**) were finally identified. The diagnostic and inclusion criteria for GBASC in our cohort is a percentage of squamous components higher than 30%, which is consistent with the diagnostic standard for ASC in pancreatic cancer in the 2010 WHO criteria (5). Tumor staging was based on the eighth American Joint Committee on Cancer (AJCC) criteria.

### Surgical procedure

All patients received a standard radical cholecystectomy, including a gallbladder incision, a partial hepatectomy, and a regional or more extended lymph node dissection. The extent of liver resection and lymphadenectomy were mainly driven by surgeons depending on the preoperative imaging, gross intraoperative findings, and intraoperative frozen biopsy. The extent of liver resection can be roughly divided into two categories: minor resection (wedge resection, SIVB + V resection) and major resection (extended hemi-hepatectomy, right or tri-segmentectomy). Lymph node dissection was performed in all patients, including regional (lymph nodes along with the cystic duct, hilum of the liver, and hepatoduodenal ligament) or extended (lymph nodes along with the pancreatic head, duodenum, and celiac artery) dissection. Node status (N0, positive lymph node = 0; N1, positive lymph nodes ≤3; and N2, positive lymph nodes >3) were evaluated based on the latest 8th AJCC criteria. When there were no enlarged lymph nodes detected pre- or intra-operatively, the surrounding fibro-fatty tissue would be cleared and submitted for pathological evaluation. Bile duct excisions were performed when obvious bile duct or hepatoduodenal ligament invasion was detected or there was intraoperatively pathologically confirmed positive cystic duct margin. When direct infiltrations of adjacent organs and structures were detected, concurrent multi-visceral resections were also performed to achieve a clear margin. R0 resection indicates a completely clear margin with no microresidual tumor. R1 resection indicates a relatively clear margin with no gross tumor or that the tumor is closely adjacent to the margin. R2 resection indicates a macroscopic residual tumor, which a specialized pathologist confirms.

### Follow up

Patients were regularly followed up *via* blood tumor biomarkers and abdominal radiological examination to detect disease progress every 1 to 2 months in the first year after surgery and every 3 to 6 months thereafter.

### Data acquisition

All relevant preoperative, intraoperative, and postoperative clinico-pathological data were retrospectively collected and recorded within a database. Preoperative details included sex (male vs female), age (≥60 vs <60), preoperative CA199 level (≥37 U/ml vs <37 U/ml), preoperative jaundice, and preoperative biliary drainage. Intraoperative details included the extent of liver resection (minor vs major), the number of harvested lymph nodes and positive lymph nodes, bile duct resection, portal vein or hepatic artery reconstruction, combined multi-visceral resection, and the R0 resection rate. The size of the resected tumor was also analyzed when provided. Postoperative details include pathologically confirmed tumor biological features (liver invasion, neural invasion, lymph-vascular invasion, node metastasis, and tumor differentiation status), tumor stage, morbidities, mortalities, postoperative chemotherapy, overall recurrence rate, and recurrence rate within 6 months.

### Statistical analysis

IBM SPSS version 22.0 (IBM SPSS, Chicago, IL, USA), R Studio 3.6.3 software, and Graph-Pad Prism 7 were used for statistical analysis. Continuous data are recorded as medians (or ranges). Categorical data are recorded as numbers (percentages). Categorical variables were evaluated *via* Chi-squared and Fisher’s exact tests. Continuous variables were analyzed using a non-parametric test. Overall survival (OS) was defined as the period from the date of receiving radical surgery to the date of death or last follow-up. Disease-free survival (DFS) was defined as the living period from the date of receiving surgery to the date of recurrence or progression. Kaplan–Meier curves were used for evaluating survival differences. The Cox-proportional hazards model was used to create a multivariate model for independent prognostic factors for survival, which were presented was the Hazard ratio (HR) with its 95% confidence interval (CI). P-values lower than 0.05 indicated the existence of statistical significance. To evaluate the solo impact of GBASC on the entire cohort, propensity score matching (PSM) was performed *via* controlling age, sex, and various independent prognostic factors that significantly influenced prognosis (R Studio 3.6.3 software, Ratio 1:1, standard deviation 0.1).

### Meta-analysis

For further validation of the differences between GBASC/SC and GBAC, we searched the following databases, including PubMed, Embase, and the Cochrane Library. RevMan5.3 software was applied in the data analysis. Eligible studies were restricted to comparative studies between GBASC/SC and GBAC. The following terms were used: gallbladder, adenosquamous, squamous, and carcinoma. Inclusion criteria: published English literature; comparative studies provided relevant survival information. Exclusion criteria: abstracts, meetings, conferences, letters, and case reports; studies that shared the same database (the study with the largest cohort would be incorporated); studies that failed to provide survival information. The HR with its 95% CI was applied in the survival analysis, and Tierney’s method would be used for a rough estimate of HR when not directly provided (11). The odds ratio (OR) was applied to dichotomous variables, and the weighted mean difference (WMD) was used for continuous variables. The method by Luo et al. was used to have a rough estimate of means and standard deviations if they were not directly provided (12). A random-effects model would be used if the heterogeneity was >50%; otherwise, a fixed-effects model would be applied.

## Results

### Baseline characteristics

**Figure 2:**
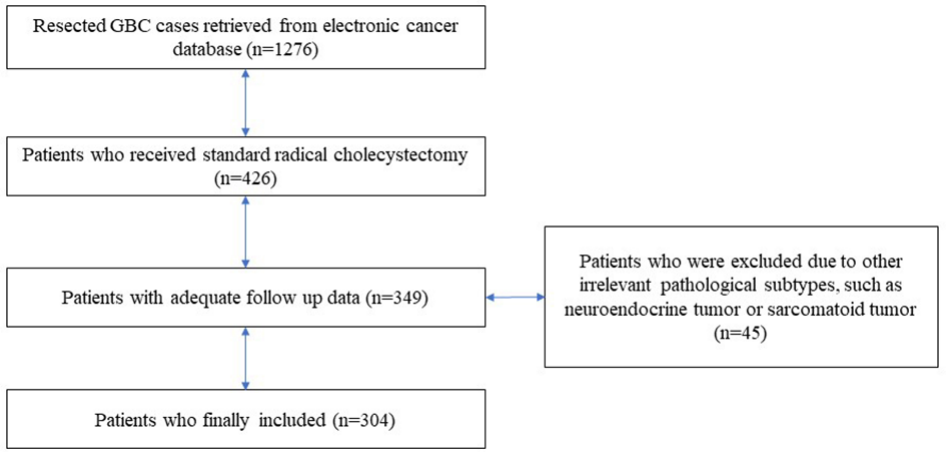
Flowchart on the specific process of patient selection and identification.

As **Figure 2** illustrates, according to the inclusion and exclusion criteria, 304 patients with post-surgical, pathologically confirmed GBC were identified, including 34 patients with GBASC and 270 patients with conventional GBAC. Ninety-nine patients were male, and 205 were female. The median age of the entire cohort was 60 (30–82). All patients received curative-intent surgery, and cases with distant metastasis were ruled out (**Table 1**).

**Table 1:** Clinico-pathological features between patients with GBASC and GBAC before and after PSM.

Variable, n (%)	Before PSM			After PSM		
	GBASC (n = 34)	GBAC (n = 270)	P-value	GBASC (n = 34)	GBAC (n = 87)	P-value
Age			P = 0.087			P = 0.479
≥60	22 (64.7%)	137 (50.7%)		22 (64.7%)	54 (62.1%)	
<60	12 (35.3%)	133 (49.3%)		12 (35.3%)	33 (37.9%)	
Sex			P = 0.286			P = 0.458
Male	13 (38.2%)	86 (31.99%)		13 (38.2%)	36 (41.4%)	
Female	21 (61.8%)	184 (68.1%)		21 (61.8%)	51 (58.6%)	
Preoperative CA199			P <0.0001			P = 0.138
≥37 U/ml	22 (64.7%)	84 (31.1%)		22 (64.7%)	45 (51.7%)	
<37 U/ml	12 (35.3%)	186 (68.9%)		12 (35.3%)	42 (48.3%)	
Preoperative jaundice	6 (17.6%)	34 (12.6%)	P = 0.278	6 (17.6%)	22 (25.3%)	P = 0.260
Preoperative biliary drainage (PTCD or ENBD)	1 (2.9%)	6 (2.2%)	P = 0.568	1 (2.9%)	6 (6.9%)	P = 0.364
Bile duct resection	7 (20.6%)	58 (21.5%)	P = 0.554	7 (20.6%)	30 (34.5%)	P = 0.100
Type of hepatectomy			P = 0.350			P = 0.369
Minor hepatectomy	5 (14.7%)	30 (11.1%)		5 (14.7%)	17 (19.5%)	
Major hepatectomy	29 (85.3%)	240 (88.9%)		29 (85.3%)	70 (80.5%)	
Combined portal vein reconstruction	3 (8.8%)	8 (3%)	P = 0.113	3 (8.8%)	4 (4.6%)	P = 0.307
Combined hepatic artery reconstruction	0 (0%)	10 (3.7%)	P = 0.300	0 (0%)	6 (6.9%)	P = 0.131
Combined multi-visceral resection*	7 (20.6%)	31 (11.5%)	P = 0.111	7 (20.6%)	19 (21.8%)	P = 0.546
Liver invasion	24 (70.6%)	102 (37.8%)	P <0.0001	24 (70.6%)	47 (54%)	P = 0.071
Neural invasion	4 (11.8%)	59 (21.9%)	P = 0.124	4 (11.8%)	27 (31%)	P = 0.022
Lymph-vascular invasion	4 (11.8%)	39 (14.4%)	P = 0.455	4 (11.8%)	18 (20.7%)	P = 0.191
Margin status			P = 0.328			P = 0.514
R0	30 (88.2%)	248 (91.9%)		30 (88.2%)	75 (86.2%)	
R1/R2	4 (11.8%)	22 (8.1%)		4 (11.8%)	12 (13.8%)	
Tumor size (continuous, cm)	3 (1–15)	3 (0.5–10)	P = 0.060	3 (1–15)	3.3 (1–10)	P = 0.493
pT (8th AJCC)			P <0.0001			P = 0.551
T1–T2	3 (8.8%)	110 (40.7%)		3 (8.8%)	9 (10.3%)	
T3–T4	31 (91.2%)	160 (59.3%)		31 (91.2%)	78 (89.7%)	
pN (8th AJCC)			P = 0.822			P = 0.521
N0	23 (67.6%)	169 (62.6%)		23 (67.6%)	49 (56.3%)	
N1	8 (23.5%)	77 (28.5%)		8 (23.5%)	28 (32.2%)	
N2	3 (8.9%)	24 (8.9%)		3 (8.9%)	10 (11.5%)	
No. retrieved lymph nodes	5 (0–16)	5 (0–21)	P = 0.650	5 (0–16)	5 (0–21)	P = 0.896
No. positive lymph nodes	0 (0–5)	0 (0–8)	P = 0.666	0 (0–5)	0 (0–8)	P = 0.279
Overall stage (8th AJCC)			P = 0.003			P = 0.798
I	0 (0%)	35 (13%)		0 (0%)	2 (2.3%)	
II	3 (8.8%)	67 (24.8%)		3 (8.8%)	7 (8%)	
III	16 (47.1%)	107 (39.6%)		16 (47.1%)	44 (50.6%)	
IV	15 (44.1%)	61 (22.6%)		15 (44.1%)	34 (39.1%)	
Differentiation status			P = 0.065			P = 0.001
low to moderate	23 (67.6%)	218 (80.7%)		23 (67.6%)	81 (93.1%)	
High	11 (32.4%)	52 (19.3%)		11 (32.4%)	6 (6.9%)	
Postoperative adjuvant chemo-radiotherapy			P = 0.075			P = 0.610
Yes	5 (14.7%)	74 (27.4%)		5 (14.7%)	13 (14.9%)	
No	29 (85.3%)	194 (82.6%)		29 (85.3%)	74 (85.1%)	
Morbidities	8 (23.5%)	42 (15.6%)	P = 0.173	8 (23.5%)	14 (16.1%)	P = 0.241
Mortalities	0 (0%)	2 (0.7%)	P = 0.788	0 (0%)	2 (2.3%)	P = 0.515
Overall Recurrence	27 (79.4%)	154 (57%)	P = 0.010	27 (79.4%)	71 (81.6%)	P = 0.386
Recurrence within 6 months	11 (32.4%)	26 (9.6%)	P = 0.001	11 (32.4%)	20 (23%)	P = 0.222

GBASC, gallbladder adenosquamous carcinoma; GBAC, gallbladder adenocarcinoma; PTCD, percutaneous trans-hepatic cholangial drainage; ENBD, endoscopic naso-biliary drainage; AJCC, American Joint Committee on Cancer; PSM, propensity score matching.

### Comparison of clinico-pathological features between patients with GBASC and GBAC

The clinico-pathological features of the entire cohort, grouped by pathological types, are summarized in **Table 1**. Regarding preoperative details, comparable sex (male vs female), age (≥60 and <60), preoperative jaundice (P = 0.278), and preoperative biliary drainage (P = 0.568) were observed between the two groups. However, patients with GBASC had a significantly higher incidence of preoperative CA199 ≥37 U/ml (P <0.0001). Regarding intraoperative details, patients with GBASC shared comparable incidences of receiving bile duct resection (P = 0.554), major hepatectomy (P = 0.350), and major vascular reconstruction (portal vein or hepatic artery) (P >0.05). Combined multi-visceral resections were more frequently performed in patients with GBASC, although the result did not reach a statistical difference (20.6% vs 11.5%, P = 0.111). A comparable R0 resection rate was also acquired (88.2% vs 91.9%, P = 0.328). Regarding postoperative pathological outcomes, patients with GBASC had comparable incidences of neural invasion (P = 0.124), lymph-vascular invasion (P = 0.455), node metastasis (P = 0.822), and well-to-moderate differentiation status (P = 0.065). A comparable number of harvested lymph nodes as well as the number of positive lymph nodes were also acquired. However, the incidence of liver invasion was significantly higher in patients with GBASC (70.6% vs 37.8%, P <0.0001). Besides, the percentage of patients with T3–4 disease (91.2% vs 59.3%, <0.0001) or III–IV disease (91.2% vs 62.2%, P = 0.003) was significantly higher in patients with GBASC. Patients with GBAC received postoperative chemotherapy more frequently than patients with GBASC (27.4% vs 14.7%, P = 0.075). Comparable incidences of overall morbidities (P = 0.173) and mortalities (P = 0.788) were acquired between two groups. Two patients died peri-operatively due to an abdominal pseudoaneurysm rupture. The overall recurrence rate (79.4% vs 57%, P = 0.010) and recurrence rate within 6 months (32.4% vs 9.6%, P = 0.001) were significantly higher in patients with GBASC (**Table 1**).

### Survival outcomes

**Figure 3:**
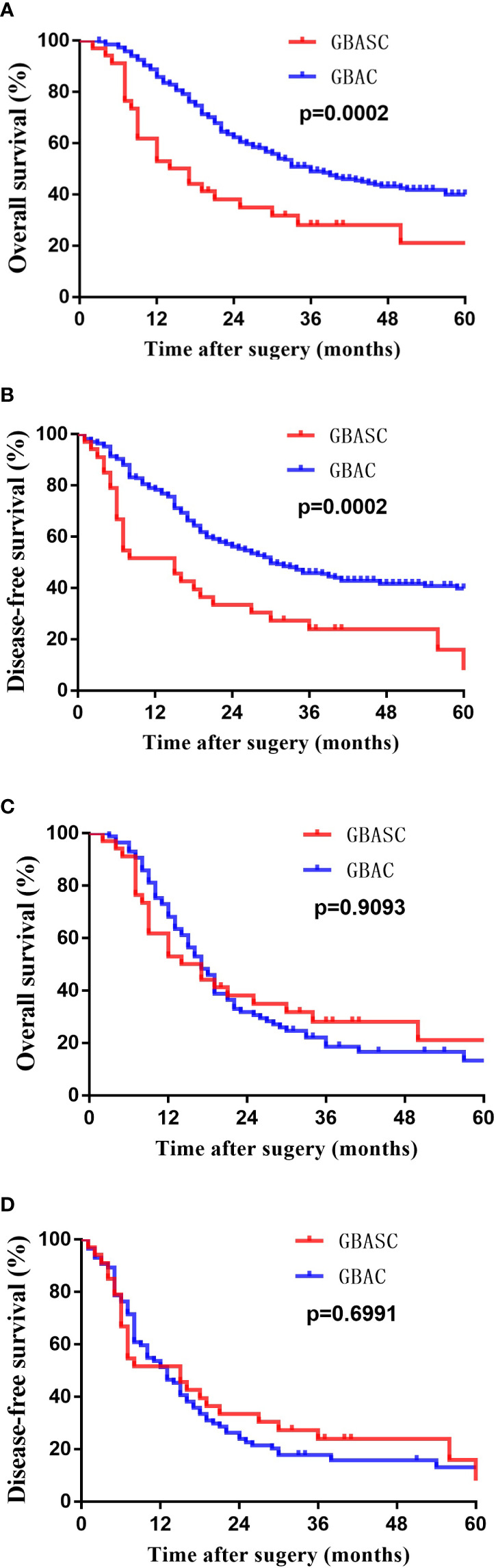
Kaplan–Meier curves for long-term survival of patients with GBASC and GBAC after curative-intent resection. **(A)** OS before PSM; **(B)** DFS before PSM; **(C)** OS after PSM; and **(D)** DES after PSM.

**Figure 4:**
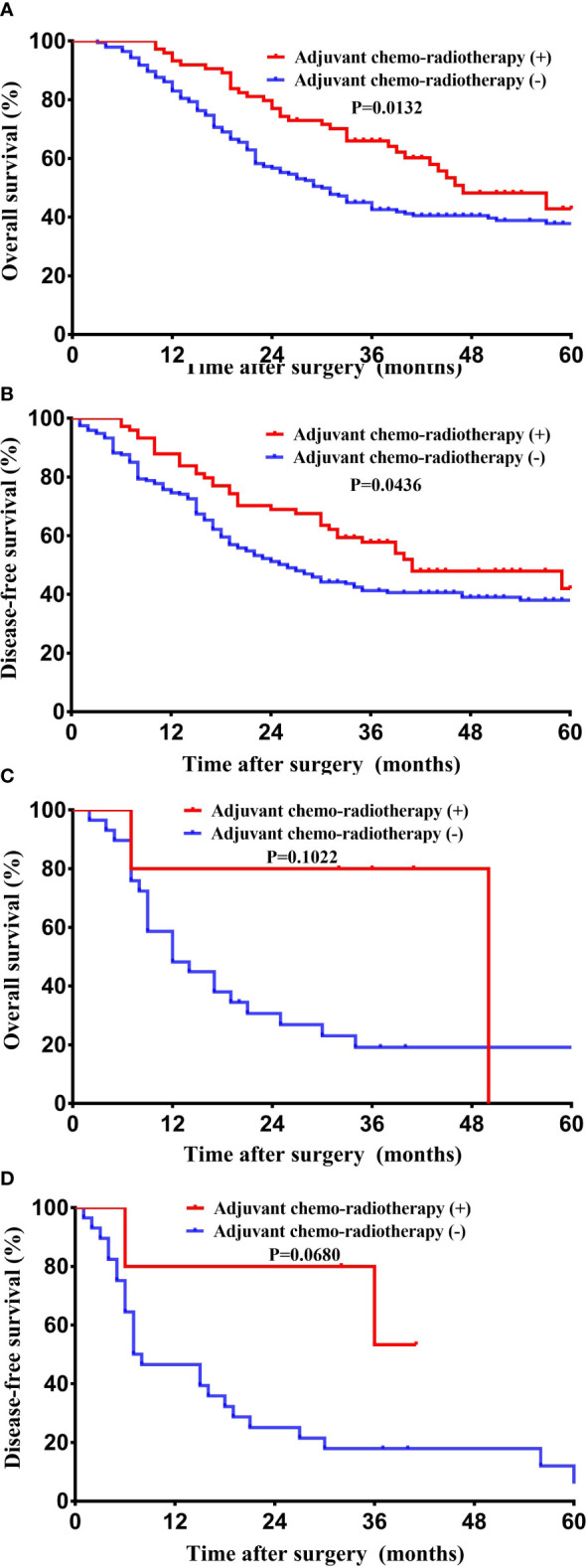
Kaplan–Meier curves for long-term survival between patients who received adjuvant chemo-radiotherapy and those without it. **(A)** OS among patients with pure GBAC; **(B)** DFS among patients with pure GBAC; **(C)** OS among patients with GBASC; and **(D)** DFS among patients with GBASC.

The median survival time of the entire cohort (n = 302; two cases of death peri-operatively were excluded) was 31 months, ranging from 2 months to 124 months. The 1-, 3-, and 5-year survival rate of the entire cohort was 85.8%, 40.4%, and 13.9%. Patients with GBASC had a significantly worse OS (median survival time: 15.5 months vs 36 months, P = 0.0002) (**Figure 3A**) as well as DFS (median recurrence time after surgery: 15 months vs 30 months, P = 0.0002) (**Figure 3B**) than patients with pure GBAC. Moreover, we also evaluated the survival impact of postoperative adjuvant chemo-radiotherapy in patients with GBAC and GBASC, respectively. Patients with GBAC could benefit from adjuvant chemo-radiotherapy. Patients who received adjuvant chemo-radiotherapy shared a better OS (median survival time: 47 months vs 30 months, P = 0.0132) (**Figure 4A**) or DFS (median survival time: 41 months vs 26 months, P = 0.0436) (**Figure 4B**) than those who did not. As for patients with GBASC, only five patients received postoperative adjuvant chemo-radiotherapy and its survival impact seemed to be vague. For patients with GBASC, comparable OS (median survival time: 50 months vs 12 months, P = 0.1022) (**Figure 4C**) and DFS (median survival time: 36 months vs 8 months, P = 0.0680) (**Figure 4D**) were obtained between those who received adjuvant chemo-radiotherapy and those who did not.

### Univariate and multi-variate analyses of prognostic factors on OS of the entire cohort

A Cox-proportional hazards regression model was used to identify the independent prognostic factors in patients with GBC. The results indicated that preoperative CA199 (≥37 U/ml vs <37 U/ml) (P <0.0001), bile duct resection (P <0.0001), major hepatectomy (P = 0.011), combined multi-visceral resection (P <0.0001), R0 resection (P <0.0001), node metastasis (P <0.0001), neural invasion (P <0.0001), lymph-vascular invasion (P <0.0001), liver invasion (P <0.0001), tumor differentiation status (low to moderate vs high) (P <0.0001), T stage (T1–2 vs T3–4, P <0.0001), pathological type (GBASC vs GBAC, P <0.0001), and postoperative adjuvant chemo-radiotherapy (P = 0.002) were prognostic factors for patients with resected GBC. The results of multivariable analysis revealed that R0 resections (P = 0.001), node metastasis (P <0.0001), T stage (T1–2 vs T3–4) (P <0.0001), and postoperative adjuvant chemo-radiotherapy (P <0.0001) were all independent prognostic factors (**Table 2**).

**Table 2:** Survival analysis, univariate, and multivariable analysis on OS of the entire cohort.

Variables	N	Survival rate		P-value, Univariate	HR (95% CI), Multivariate	P-value, Multivariate
		3-year survival rate	5-year survival rate			
Sex				P = 0.990	–	–
Male	97	39.20%	9.30%			
Female	205	41.00%	16.10%			
Age (years)				P = 0.114	–	–
≥60	158	34.80%	12.7%			
<60	144	46.50%	15.30%			
CA199 (U/ml)				P <0.0001	1.227 (0.864–1.744)	P = 0.253
≥37 U/ml	105	17.10%	5.70%			
<37 U/ml	197	52.80%	18.30%			
Bile duct resection				P <0.0001	1.173 (0.757–1.818)	P = 0.476
Performed	64	23.40%	6.30%			
Not performed	238	45.00%	16.00%			
Type of hepatectomy				P = 0.011	1.075 (0.679–1.710)	P = 0.758
Major	34	29.40%	5.90%			
Minor	268	41.80%	14.90%			
Combined multi-visceral resections				P <0.0001	0.814 (0.495–1.339)	P = 0.418
Performed	36	19.40%	6%			
Not performed	266	43.2%	15.00%			
Surgical margins				P <0.0001	2.303 (1.417–3.742)	P = 0.001
R0	25	8.00%	0.00%			
R1/R2	277	43.30%	15%			
Lymph node status				P <0.0001	1.826 (1.411–2.362)	P <0.0001
N0	191	56.00%	20.40%			
N1	84	14.3%	2.40%			
N2	27	11.10%	3.70%			
Neural invasion				P <0.0001	0.956 (0.633–1.443)	P = 0.830
Yes	62	21.00%	3.20%			
No	240	45.40%	16.70%			
Lymph-vascular invasion				P <0.0001	1.176 (0.765–1.808)	P = 0.461
Yes	42	19%	7.10%			
No	260	43.80%	15.00%			
Liver invasion				P <0.0001	0.996 (0.680–1.459)	P = 0.984
Yes	124	18.50%	4.80%			
No	178	55.60%	20.20%			
Tumor differentiation status				P <0.0001	0.870 (0.538–1.406)	P = 0.569
Low to moderate	239	35.10%	11.30%			
High	63	60.30%	23.80%			
T stage				P <0.0001	5.371 (3.133–9.209)	P <0.0001
T1–2	113	75.20%	29.20%			
T3–4	189	19.60%	4.80%			
Pathological type				P <0.0001	1.156 (0.912–1.465)	P = 0.230
GBAC	34	24%	9%			
GBASC	268	42.50%	14.60%			
Morbidities				P = 0.665	–	–
Yes	48	41.7%	4.20%			
No	254	40.20%	15.70%			
Postoperative adjuvant chemo-radiotherapy				P = 0.002	0.496 (0.335–0.736)	P <0.0001
Yes	79	54.40%	8.90%			
No	223	35.40%	15.70%			

N, number; HR, hazard ratio; CI, confidence interval; GBASC, gallbladder adenosquamous carcinoma; GBAC, gallbladder adenocarcinoma.

### Propensity score matching analysis

Considering the impact on survival brought by the inherent bias between two groups, a PSM was performed by controlling the following factors: age, sex, and acquired independent prognostic factors for OS.

After matching, as illustrated in **Table 1**, 34 patients with GBASC and 87 patients with GBAC were identified. Comparable surgical margins, tumor stage, node status, and the proportion of patients receiving postoperative chemotherapy were collected. However, neural invasion (31% vs 11.8%, P = 0.022) was more frequently detected in patients with GBAC. Moreover, a significantly higher percentage of patients with low to moderate differentiation status was also observed in patients with GBAC (93.1% vs 67.6%, P = 0.001). Comparable overall recurrence rates (P = 0.386) and the recurrence rate within 6 months (P = 0.222) were also determined. Moreover, comparable OS (median survival time: 15.5 months vs 17 months, P = 0.9093) (**Figure 3C**) and DF (median survival time: 15 months vs 13 months, P = 0.6991) (**Figure 3D**) were found between patients with GBASC and GBAC.

### Meta-analysis

According to the inclusion criteria mentioned above, six comparative studies were finally incorporated (7–9, 13–15). As summarized in **Table S1**, with our own cohort incorporated, 1,434 patients with GBASC/SC and 29,767 patients with pure GBAC were included. The primary endpoint of our additional meta-analysis was the survival difference between patients with GBASC/SC and GBAC. As summarized in **Table S2**, pooled results revealed that patients with GBASC/SC had significantly worse OS (HR = 2.27, 95% CI 1.80 to 2.85, P <0.00001) (**Figure S2A**) and DFS (HR = 2.76, 95% CI 1.81 to 4.20, P <0.00001) (**Figure S2B**) versus patients with pure GBAC. Moreover, we also analyzed the inconsistencies in the clinico-pathological features between GBASC/SC and GBAC. As illustrated in **Figure S1**, patients with GBASC/SC had a significantly larger tumor size (WMD = 1.41, 95% CI 0.44–2.37, P = 0.004) (**Figure S1B**), a significantly higher incidence of node metastasis (24.9% vs 19.5%, P = 0.02) (**Figure S1C**), a significantly higher incidence of liver involvement (78.3% vs 61.3%, P = 0.0001) (**Figure S1F**), and a significantly lower R0 rate (59.1% vs 67.2%, P <0.00001) (**Figure S1A**). Patients with GBASC/SC were more frequently in an advanced stage that the proportion of patients with T3–4 or III–IV disease were significantly higher in the GBASC/SC group (65.6% vs 52.5%, P <0.0001) (**Figure S1G**). The incidences of neural invasion (P = 0.76) (**Figure S1D**) and lymph-vascular invasion (P = 0.55) (**Figure S1E**) were comparable between the two groups.

## Discussion

GBASC is a rare but aggressive subtype of GBC, and such a rare entity is still poorly understood. In the last decade, prior to our study, numerous retrospective studies focused on the significance of the squamous component in patients with GBC. Their results consistently indicated that the co-existence of the squamous component in patients with GBASC resulted in more aggressive tumor biological features and a worse prognosis versus those with pure AC (7–9, 13–15).

Kim et al. first analyzed the survival difference between 16 patients with GBASC/SC and 360 patients with pure GBAC and found significantly worse survival in the former group (P <0.001) (7). Their observations indicated that GBC patients with a squamous component were often diagnosed at an advanced stage with a significantly lower R0 resection rate (P = 0.022). However, after matching the overall stage of both groups, a similar DFS was observed between the two groups when a negative margin was obtained (7). A similar but worse prognosis was also reported by other authors (9, 14–16). However, Leigh et al. revealed that even when negative margins were achieved, pure SC still had a worse prognosis than pure GBAC (8). Recently, a study by Ayabe et al. with the largest cohort included demonstrated that GBASC/SC tended to have a significantly lower R0 resection rate (P <0.001) and worse prognosis even after R0 resections (P <0.001) versus GBAC (13).

In line with our findings, patients with GBASC in our cohort had a relatively larger tumor size (P = 0.060) and were in a more advanced stage of the disease than patients with GBAC. The proportion of patients with T3–4 disease (91.2% vs 59.3%, P <0.0001) or patients with III–IV disease (91.2% vs 62.2%, P <0.0001) was significantly higher in patients with GBASC than in those with pure GBAC. Liver invasion was more frequently detected in patients with GBASC (P <0.0001), and the R0 resection rate was relatively lower in patients with GBASC (88.2% vs 91.9%, P = 0.328). Moreover, the proportion of patients receiving combined multi-visceral resection was significantly higher in patients with GBASC, with a borderline P-value (20.6% vs 11.5%, P = 0.111). The inconsistencies of tumor biological features, tumor stage, and the extent of resections mentioned above can partially illustrate the worse survival as well as a higher recurrence rate in patients with GBASC. Lymph node metastasis has been proven to be an independent prognostic factor for patients with GBC (17). A negative margin (R0) has also been validated as an independent prognostic factor for patients with GBC (17, 18). However, both of these vital prognostic factors were comparable between the two groups in our study. Therefore, for further validation of the significance of the squamous component in patients with GBC, univariate and multivariable analyses and a propensity score matching analysis were performed.

The results of univariate analyses indicated that pathological type can be regarded as a prognostic factor (P <0.0001). However, when pooled together with other factors, the impact of pathological type on the OS can be neglected, and many other factors, including tumor T stage (P <0.0001), N stage (P <0.0001), surgical margins (P = 0.001), and postoperative chemotherapy (P <0.0001), have a significant impact on the OS. The proportion of patients receiving postoperative adjuvant chemo-radiotherapy was significantly higher in patients with GBASC, and postoperative chemotherapy has been demonstrated as an independent prognostic factor in our cohort (P <0.0001), which has also been validated in many previously published studies (19–21). Consequently, we next performed a PSM by controlling age, sex, and the four most influential factors as mentioned above. The results revealed equivalent OS (P = 0.9093) and DFS (P = 0.6991) between the two groups. Combined with observations reported in previous studies (7, 16), we can conclude that it is tumor stage rather than pathological subtypes that directly determine prognosis. Tumor pathological subtypes are more often correlated with different tumor biological features, which partially reflect different levels of aggressiveness of the tumor and finally influence the overall tumor stage. Finally, with our results incorporated, a meta-analysis was performed on 1,434 patients with GBASC/SC and 29,767 patients with pure GBAC. Pooled results revealed significantly worse OS (P <0.00001) or DFS (P <0.00001) in patients with GBASC/SC, which further validated our findings. Moreover, regarding pooled results of tumor biological features, patients with GBASC/SC tended to have a larger tumor size (P = 0.004), a higher incidence of node metastasis (P = 0.02), a higher incidence of liver invasion (P = 0.0001), and a higher proportion of advanced disease (T3–4 or III–IV) (P <0.0001), which further explained the worse prognosis in patients with GBASC/SC.

Our study with a relatively large cohort not only revealed the inconsistencies between patients with GBASC and GBAC in terms of biological features and long-term survival but also first performed a PSM to control inherent bias. Moreover, a meta-analysis was also performed for further validation of our results and conclusions. However, there are still some limitations in our manuscript. First, only 34 patients who underwent curative surgery were identified, so the sample size is relatively small. Second, the inconsistency in the diagnostic and inclusive criteria would also introduce bias. However, it has not reached consensus on the diagnostic criteria for GBASC. Third, many other uncontrolled factors, such as the patients’ preoperative health condition, may also influence the prognosis. Fourth, a rough estimate of HR *via the* Tierney method and a rough estimate of means and standard deviations *via the* method by Luo et al. may also introduce bias to some extent. Additionally, the effect of postoperative adjuvant chemo-radiotherapy in patients with GBASC was still under exploration because only five patients with GBASC in our cohort received adjuvant chemo-radiotherapy.

## Conclusion

Our study provided a single-institute experience of the similarities and differences between patients with GBASC and GBAC. Patients with GBASC had more aggressive tumor biological features and a worse prognosis than patients with pure GBAC. Based on our findings, a meta-analysis was also performed with an extremely large sample size included, which greatly validated our results and conclusions.

## Data availability statement

The raw data supporting the conclusions of this article will be made available by the authors, without undue reservation.

## Ethics statement

Our study was approved by the ethics committee of West China Hospital. Written informed consent for participation was not required for this study in accordance with the national legislation and the institutional requirements. Written informed consent was obtained from the individual(s) for the publication of any potentially identifiable images or data included in this article.

## Author contributions

T-RL and FL contributed equally to the study. T-RL contributed to data acquisition and drafted the manuscript. FL contributed to the literature review, manuscript editing, and subsequent minor revision. Z-YL, R-QZ, W-JM, and H-JH were involved in editing the manuscript. F-YL contributed to the study design and revision of the manuscript. All authors listed have made a substantial, direct, and intellectual contribution to the work and approved it for publication.
