# Supplementary material for: Comparison of clinicopathological characteristics and long-term survival between patients with gallbladder adenosquamous carcinoma and pure gallbladder adenocarcinoma after curative-intent surgery: a single-center experience in China and a meta-analysis
Source: Front Oncol. 2023 May 2;13:1116275. doi: 10.3389/fonc.2023.1116275 (PMC10185816; doi:10.3389/fonc.2023.1116275)
Supplement: Supplementary file 1 [file DataSheet_1.pdf]

Table S1 Baseline characteristics of all studies included

Author	Study period	patient source	The inclusion criteria of GBASC	No. patients		Follow up (months)	Quality Score (NOS)
				GBASC/GBSC	GBAC		
Roa JC et al 2011	NA	Temuco, Chile; Wayne State University, Emory University, America	squamous component≥30%	34	572	NA	6
Kim WS et al 2011	1994-2009	Samsung Medical Center, Korea	squamous component≥30%	16	360	Median 6, range (3.8-126.9)	7
Song HW et al 2015	2008-2012	Affiliated Hospital of Xi'an Jiao Tong University, China	squamous component≥30%	34	377	Median 21	7
Samuel S et al 2017	1988-2009	SEER	NA	217	4154	NA	6
Leigh N et al 2020	2007-2017	Mount Sinai healthcare system, America	NA	15	76	Median 17	7
Ayabe RI et al 2021	2004-2015	NCDB	NA	1084	23958	NA	6
Lv TR et al 2022*	2010-2021	West China Hospital, Sichuan university, China	squamous component≥30%	34	270	One to two months in the first year and three to six months thereafter	7

No.: the number of; GBASC: gallbladder adeno-squamous carcinoma; SC: squamous carcinoma; GBAC: gallbladder adenocarcinoma; NOS: Newcastle Ottawa Scale; NA: not available; SEER: The Surveillance, Epidemiology, and End Results Program of the National Cancer Institute; NCDB: national cancer database; *: ours

Table S2 Pooled results of all available studies in measured outcomes

Outcomes	No. studies	No. patients		OR/HR/WMD	95% CI	P value
		GBASC/SC	GBAC			
**OS**	7	1101	22454	HR=2.27	1.80-2.85	P<0.00001
**DFS**	4	272	4534	HR=2.76	1.81-4.2	P<0.00001
**R0**	6	756	15756	OR=0.69	0.60-0.81	P<0.00001
**Tumor size (continuous, cm)**	4	1149	24336	WMD=1.41	0.44-2.37	P=0.004
**Node metastasis**	6	1349	27993	OR=1.18	1.03-1.34	P=0.02
**Neural invasion**	3	83	396	OR=0.85	0.29-2.45	P=0.76
**Lymph-vascular invasion**	2	49	346	OR=1.45	0.43-4.89	P=0.55
**Liver invasion**	3	83	723	OR=3.11	1.75-5.51	P=0.0001
**T3-4/III-IV disease**	7	1434	29455	OR=2.27	1.54-3.36	P<0.0001

OS: overall survival; DFS: disease-free survival; No.: the number of; GBASC: gallbladder adeno-squamous carcinoma; GBAC: gallbladder adenocarcinoma; OR: odds ratio; HR: hazard ratio; WMD: weighted mean difference; CI: confidence interval.

Figure S1

Figure S1A

	GBASC/SC		GBAC			Odds Ratio
Study or Subgroup	Events	Total	Events	Total	Weight	M-H, Fixed, 95% CI
Ayabe RI et al 2021	384	657	10112	15001	91.2%	0.68 [0.58, 0.80]
Kim WS et al 2010	8	16	26	32	2.2%	0.23 [0.06, 0.87]
Leigh N et al 2020	11	15	50	76	1.1%	1.43 [0.41, 4.93]
Lv TR et al 2022	30	34	248	270	1.7%	0.67 [0.21, 2.06]
Song HW et al 2015	14	34	150	377	3.8%	1.06 [0.52, 2.16]
**Total (95% CI)**		**756**		**15756**	**100.0%**	**0.69 [0.60, 0.81]**
Total events	447		10586			

Heterogeneity: Chi² = 5.39, df = 4 (P = 0.25); I² = 26%
Test for overall effect: Z = 4.76 (P < 0.00001)

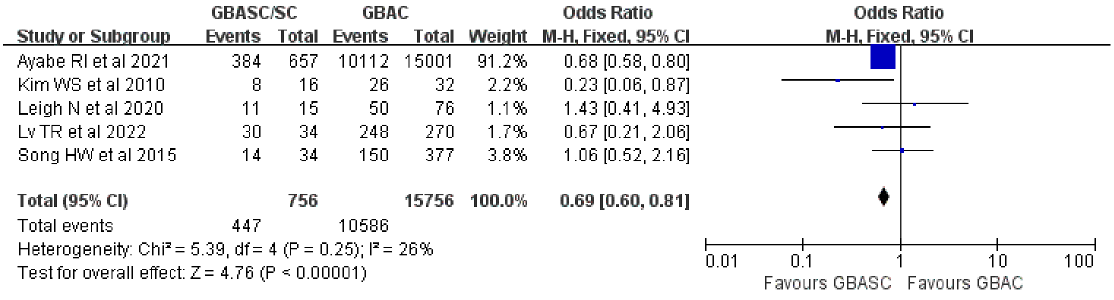

Figure S1B

	GBASC/SC			GBAC				Std. Mean Difference
Study or Subgroup	Mean	SD	Total	Mean	SD	Total	Weight	IV, Random, 95% CI
Ayabe RI et al 2021	5.4	3.7	1084	3.4	2.2	23958	27.0%	0.88 [0.81, 0.94]
Kim WS et al 2010	5.2	2	16	3.5	1.4	32	24.2%	1.03 [0.39, 1.67]
Leigh N et al 2020	5.9	1.3	15	2.8	0.6	76	22.6%	4.07 [3.25, 4.89]
Lv TR et al 2022	3.1	3.4	34	3.1	1.7	270	26.1%	0.00 [-0.36, 0.36]
**Total (95% CI)**			**1149**			**24336**	**100.0%**	**1.41 [0.44, 2.37]**

Heterogeneity: Tau² = 0.90; Chi² = 81.74, df = 3 (P < 0.00001); I² = 96%
Test for overall effect: Z = 2.86 (P = 0.004)

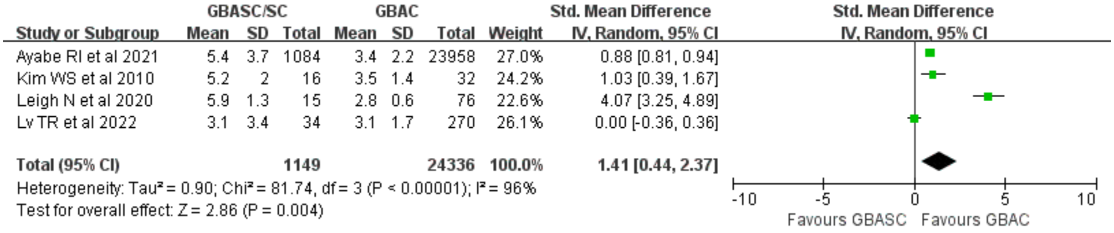

Figure S1C

	GBASC/SC		GBAC			Odds Ratio
Study or Subgroup	Events	Total	Events	Total	Weight	M-H, Fixed, 95% CI
Ayabe RI et al 2021	233	1084	4495	23958	76.8%	1.19 [1.02, 1.38]
Kim WS et al 2010	13	16	15	32	0.5%	4.91 [1.17, 20.62]
Leigh N et al 2020	9	15	24	76	0.8%	3.25 [1.04, 10.17]
Lv TR et al 2022	11	34	101	270	3.8%	0.80 [0.37, 1.71]
Samuel S et al 2017	43	166	874	3280	15.7%	0.96 [0.67, 1.37]
Song HW et al 2015	27	34	274	377	2.3%	1.45 [0.61, 3.43]
**Total (95% CI)**		**1349**		**27993**	**100.0%**	**1.18 [1.03, 1.34]**
Total events	336		5783			

Heterogeneity: Chi² = 9.31, df = 5 (P = 0.10); I² = 46%
Test for overall effect: Z = 2.43 (P = 0.02)

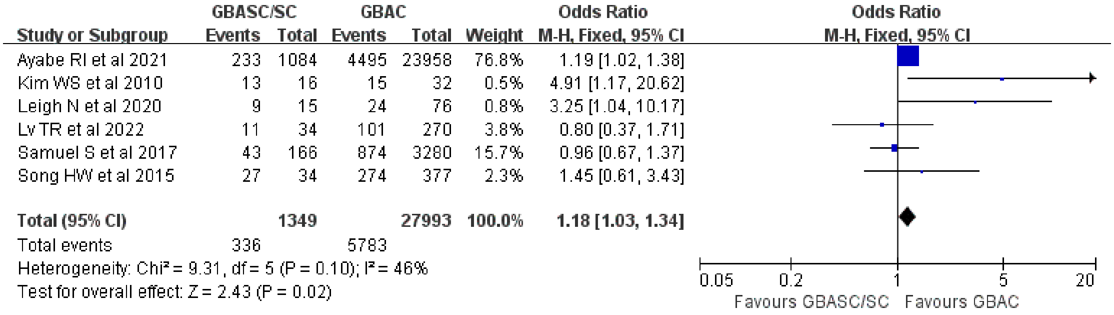

Figure S1D

	GBASC/SC		GBAC			Odds Ratio
Study or Subgroup	Events	Total	Events	Total	Weight	M-H, Random, 95% CI
Leigh N et al 2020	9	15	27	76	31.4%	2.72 [0.88, 8.47]
Lv TR et al 2022	4	34	59	270	32.5%	0.48 [0.16, 1.41]
Roa JC et al 2011	11	34	24	50	36.1%	0.52 [0.21, 1.28]
**Total (95% CI)**		**83**		**396**	**100.0%**	**0.85 [0.29, 2.45]**
Total events	24		110			

Heterogeneity: Tau² = 0.59; Chi² = 6.24, df = 2 (P = 0.04); I² = 68%
Test for overall effect: Z = 0.30 (P = 0.76)

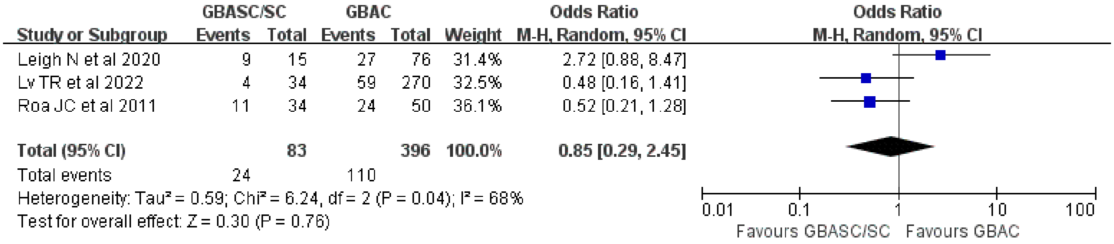

Figure S1E

Study or Subgroup	GBASC/SC Events	GBASC/SC Total	GBAC Events	GBAC Total	Weight	Odds Ratio M-H, Random, 95% CI
Leigh N et al 2020	9	15	27	76	49.3%	2.72 [0.88, 8.47]
Lv TR et al 2022	4	34	39	270	50.7%	0.79 [0.26, 2.37]
**Total (95% CI)**		**49**		**346**	**100.0%**	**1.45 [0.43, 4.89]**
Total events	13		66			

Heterogeneity: Tau² = 0.44; Chi² = 2.37, df = 1 (P = 0.12); I² = 58%
Test for overall effect: Z = 0.60 (P = 0.55)

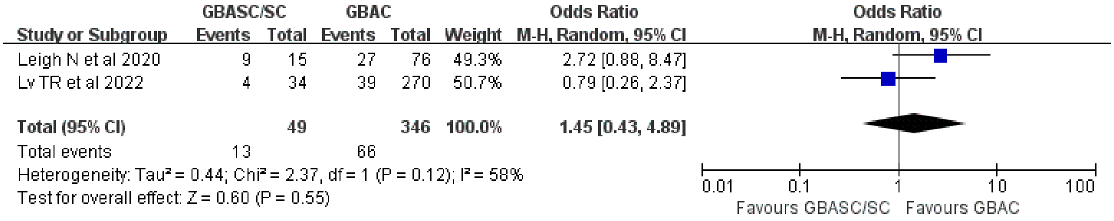

Figure S1F

Study or Subgroup	GBASC/SC Events	GBASC/SC Total	GBAC Events	GBAC Total	Weight	Odds Ratio M-H, Fixed, 95% CI
Leigh N et al 2020	11	15	28	76	16.1%	4.71 [1.37, 16.22]
Lv TR et al 2022	30	34	313	377	39.9%	1.53 [0.52, 4.50]
Song HW et al 2015	24	34	102	270	44.0%	3.95 [1.82, 8.60]
**Total (95% CI)**		**83**		**723**	**100.0%**	**3.11 [1.75, 5.51]**
Total events	65		443			

Heterogeneity: Chi² = 2.46, df = 2 (P = 0.29); I² = 19%
Test for overall effect: Z = 3.89 (P = 0.0001)

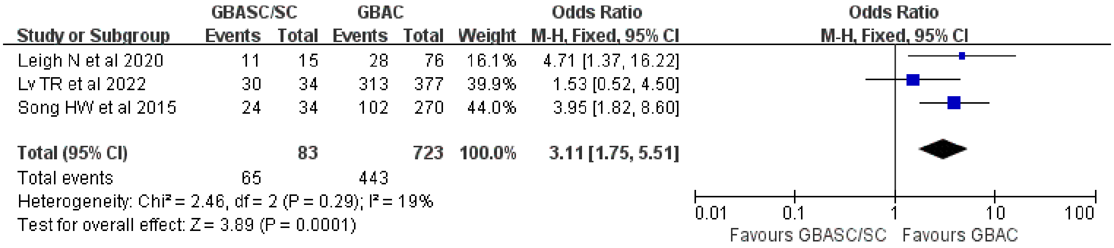

Figure S1G

Study or Subgroup	GBASC/SC Events	GBASC/SC Total	GBAC Events	GBAC Total	Weight	Odds Ratio M-H, Random, 95% CI
Ayabe RI et al 2021	687	1084	12763	23958	34.5%	1.52 [1.34, 1.72]
Kim WS et al 2010	16	16	19	48	1.8%	49.92 [2.83, 881.42]
Leigh N et al 2020	11	15	40	76	7.9%	2.48 [0.72, 8.47]
Lv TR et al 2022	31	34	168	270	8.1%	6.27 [1.87, 21.05]
Roa JC et al 2011	23	34	280	572	15.8%	2.18 [1.04, 4.56]
Samuel S et al 2017	139	217	1848	4154	30.1%	2.22 [1.67, 2.95]
Song HW et al 2015	34	34	359	377	1.8%	3.55 [0.21, 60.21]
**Total (95% CI)**		**1434**		**29455**	**100.0%**	**2.27 [1.54, 3.36]**
Total events	941		15477			

Heterogeneity: Tau² = 0.11; Chi² = 17.38, df = 6 (P = 0.008); I² = 65%
Test for overall effect: Z = 4.10 (P < 0.0001)

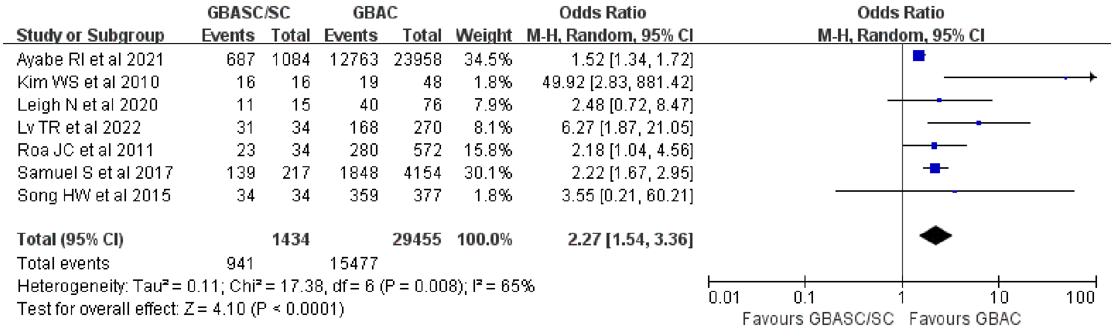

Figure S1 Forest plots presenting the tumor biological features between patients with GBASC/SC and GBAC. A, R0 resection rate; B, tumor size (continuous, cm); C, node metastasis; D, neural invasion; E, lymph-vascular invasion; F, liver invasion; G, the proportion of patients with T3-4 or III-IV disease.

Figure S2

Figure S2A

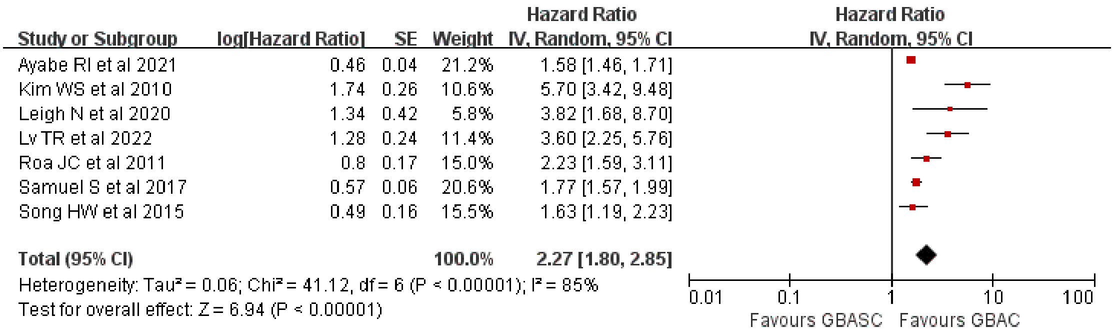

Figure S2B

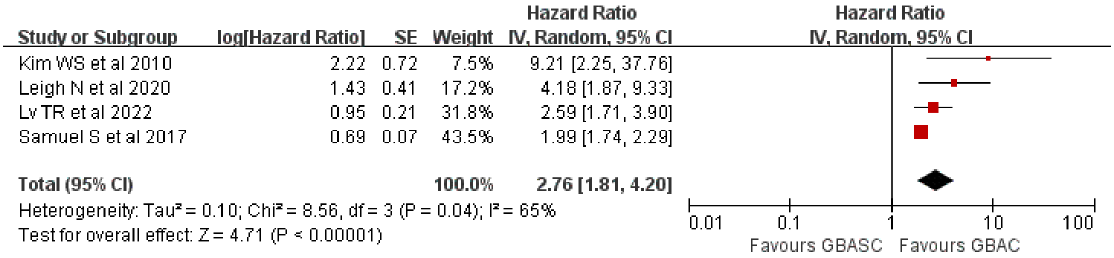

Figure S2 Forest plots presenting survival outcomes in patients with GBASC/SC and GBAC. A, OS; B, DFS.
